# Dentoalveolar Changes in the Premolar Region After Two Maxillary Expansion Protocols

**DOI:** 10.1111/ocr.70122

**Published:** 2026-04-10

**Authors:** Jaqueline Peter, Fabiane Azeredo, Susana Maria Deon Rizzatto, Cátia Cardoso Abdo Quintão, Tarek Elshebiny, Juan Martin Palomo, Luciane Macedo de Menezes

**Affiliations:** ^1^ Department of Orthodontics, School of Health and Life Sciences (Dental Program) Pontifical Catholic University of Rio Grande Do Sul Porto Alegre Brazil; ^2^ Department of Preventive and Community Dentistry, School of Dentistry State University of Rio de Janeiro Rio de Janeiro Brazil; ^3^ Department of Orthodontics Case Western Reserve University Cleveland Ohio USA

**Keywords:** cone‐beam CT, orthodontics, palatal expansion technique, premolars

## Abstract

**Objectives:**

To evaluate the dentoalveolar effects on the upper first premolars after two maxillary expansion protocols—rapid maxillary expansion (RME) and alternate rapid maxillary expansion and constriction (Alt‐RAMEC)—before and 6 months after treatment.

**Materials and Methods:**

Cone‐Beam Computed Tomography (CBCT) images of 20 patients who underwent maxillary expansion with the Hyrax appliance under two activation protocols were evaluated. Dental and alveolar measurements at the premolar region (tooth length, alveolar bone level and thickness) were measured before (T1) and after expansion (T2). Intra‐ and inter‐examiner reliability was determined using Intraclass Correlation Coefficients. Statistical analyses included the Shapiro–Wilk test and Student's *t‐test*s for paired and independent samples. Statistical significance was set at 5%.

**Results:**

A significant increase in interpremolar distances was observed, both in the radicular and coronal levels (4.29 mm and 4.97 mm), with greater coronal than root distance. This was more evident in the Alt‐RAMEC group. Alveolar thickness showed an average reduction of 0.54 mm after expansion.

**Conclusions:**

The expansion effects of the two protocols were similar in the premolar region, resulting in a reduction in alveolar thickness. A tendency for greater tooth inclination and root reduction was observed after the Alt‐RAMEC protocol.

**Trial Registration:**

IRB: 42856915.1.0000.5336

## 1 Introduction

Transverse maxillary deficiency is a common clinical problem affecting individuals of various age groups [1, 2]. This condition is often associated with other dentofacial deformities, resulting in both functional and aesthetic alterations. Severe transverse maxillary deficiency can cause dental malocclusion and adverse periodontal responses [1]. Crossbite, when left untreated, can lead to asymmetrical facial growth [3], functional deviations and jaw position discrepancies [4]. Maxillary expansion is the recommended treatment for growing patients with transverse deficiencies of the upper arch, as well as arch perimeter discrepancies that require suture disarticulation to facilitate maxillary protraction [5, 6]. Activation of the expansion device can occur through conventional rapid maxillary expansion (RME), slow expansion or alternate rapid maxillary expansion and constriction (Alt‐RAMEC), as proposed by Liou and Tsai [6], to promote greater suture disarticulation.

RME, as described by the classic studies of Haas [7, 8, 9, 10], produces significant skeletal and dental effects through the opening of the midpalatal suture and the lateral displacement of the maxillary halves. The expansion promotes a triangular separation of the suture, with a greater opening anteriorly and diminishing posteriorly. This results in an increase in maxillary basal width and a widening of the nasal cavity, which can improve nasal airflow [7, 8]. The orthopaedic forces also influence adjacent circummaxillary sutures, contributing to broader facial structural adaptations [9]. In the dental level, RME increases intermolar and intercanine widths, with buccal tipping of the upper posterior teeth, alveolar bending and remodelling as the dentoalveolar complex adapts to the applied forces. The skeletal and dental changes effectively correct transverse maxillary deficiencies and posterior crossbites while establishing a more favourable environment for occlusal development [7, 8, 9].

The Alt‐RAMEC produces significant skeletal and dental effects as well, including buccal and lingual tipping of posterior teeth and dentoalveolar remodelling. In theory, the repeated weekly protocol of expansion and constriction loosens the circummaxillary sutures more effectively than a single expansion phase, as RME does, thereby leading to a greater mobilization of the maxillary complex and facilitating orthopaedic displacement when combined with extra‐oral appliances [6].

Maxillary expansion occurs when the force applied to the teeth and jaw exceeds the limits necessary for tooth movement, resulting in orthopaedic correction. This process creates hyalinization of the periodontal ligament in the anchor teeth, transferring the forces to the midpalatal suture to achieve a more orthopaedic and less orthodontic effect [9]. The applied force promotes the widening and gradual opening of the median palatal, circumzygomatic and circummaxillary sutures, along with compression of the periodontal ligament, changes in the alveolar cortex and tooth inclination [11, 12].

The Hyrax‐type appliance is one of the most commonly used devices for correcting crossbites in deciduous, mixed and young permanent dentitions. This appliance is tooth‐supported, consisting of an expander screw and metal extensions welded to bands that are cemented to deciduous molars or premolars and to the first permanent maxillary molars [13]. Due to its tooth‐supported anchorage, the forces are primarily transmitted to the supporting teeth and their buccal bone walls. The orthopaedic and dentoalveolar changes promoted can be analysed using Cone‐Beam Computed Tomography (CBCT). To our knowledge, there are no CBCT studies in the literature primarily evaluating the effects of different expansion protocols with the same appliance design on the premolars, and only a few on the molars anchor teeth [14, 15, 16].

The null hypothesis of the present study was that there would be no differences between the two expansion protocols (RME and Alt‐RAMEC) on dentoalveolar changes in the premolar region. Thus, the objective of this study was to evaluate the effects on the upper first premolars and supporting structures (tooth length, alveolar bone level and thickness) after two maxillary expansion protocols (RME and Alt‐RAMEC), both before and 6 months after treatment.

## 2 Material and Methods

The study was approved by the ethical committee of the Pontifical Catholic University of Rio Grande do Sul (PUCRS, Brazil). A sample of growing patients undergoing maxillary expansion was selected from the database of previous randomized clinical trials in the Department of Orthodontics at the School of Health and Life Science at PUCRS. Informed consent was obtained from both patients and their parents, who agreed to participate in the research. For the purposes of the present investigation, cases were retrospectively selected and analysed exclusively during the expansion phase, prior to any orthopaedic protraction. In all included patients, transverse maxillary deficiency was the primary indication for expansion.

Cases included in the present study were required to meet the following inclusion criteria: presence of transverse maxillary deficiency treated with a Hyrax expander supported by four bands (anchored in first molars and first premolars), late mixed or early permanent dentition, with fully erupted upper first premolars and molars and availability of good quality CBCT images before expansion (T1) and 6 months after expansion (T2). The exclusion criteria included dental agenesis, congenital craniofacial malformations, periodontal disease and previous surgical or other treatment that could interfere with the expansion effects during the treatment period (e.g., appliance breakage, inadequate activation, use of facemask or headgears). Cases with poor‐quality CBCT scans, artefacts, or image distortions were also excluded.

Of the original database of 80 individuals, 20 met the inclusion and exclusion criteria for this research, with the two activation protocols studied (RME and Alt‐RAMEC). Thus, the convenience sample consisted of 20 individuals (13 males, seven females; 40 premolars), who underwent two maxillary expansion protocols with the Hyrax‐type expander. This device consisted of an 11 mm expansion screw (Morelli, Sorocaba, São Paulo, Brazil), four bands (upper permanent first molars and first premolars), buccal bars (1.0 mm) and palatine bars (1.4 mm) adapted and welded directly to the bands. All the CBCT images (DICOM format) were obtained from an i‐CAT scanner (Imaging Sciences International, Hatfield, Pa) at 120 kV, 8 mA, scanning time of 40 s and 0.3 mm voxel dimension [17].

For the RME group, the expansion screw was activated to 0.8 mm (4/4 turns) on the first day. From the second day onward, a daily activation of 1/4 of a turn was carried out in the morning and 1/4 at night, for a daily total of 2/4 of a turn, with a 0.4 mm daily opening. The screw was activated until it reached 8.0 mm of opening. The activation protocol for the Alt‐RAMEC group was similar: 0.8 mm (4/4 turns) on the first day and 2/4 turns per day thereafter. Patients were instructed to perform activation for a week and deactivation (closing the screw) at the same daily rate over the next week. The expansions and constrictions were repeated for 7 weeks [6], with the last activation in an opening direction, resulting in a total of 6.4 mm of screw opening. Overcorrection of the transverse dentoskeletal discrepancy in all groups was achieved [15]. Expansion retention was performed with the maintenance of the Hyrax appliance, stabilizing the screw with a 0.30″ ligature wire for 6 months, when the control CBCT was obtained.

### 2.1 CBCT Evaluation

DICOM images were analysed in the axial, coronal and sagittal views using the InVivo5 software program (Anatomage Dental, San Jose, California, USA). The effects of expansion in the upper first premolar region were evaluated at two time points: T1 (pre‐expansion) and T2 (6 months after expansion) for the two protocols evaluated (RME and Alt‐RAMEC). The tomographic views were standardized using the root of the upper first premolars (teeth 14 and 24) or the buccal root (when there were two roots) of the same teeth. The measurements were standardized and applied across all patients.

### 2.2 Alveolar Measurements—Upper First Premolar Region

**FIGURE 1:**
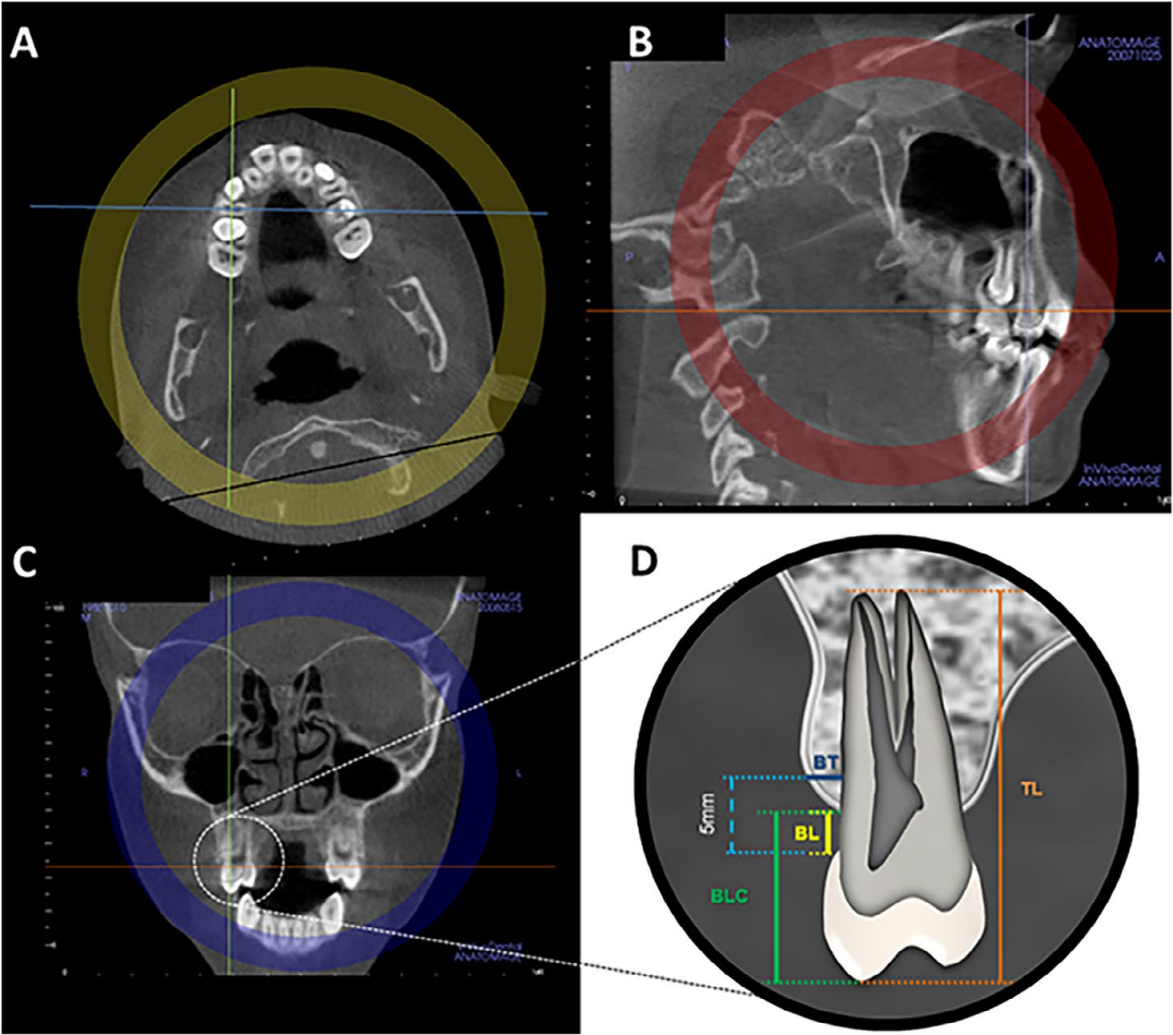
CBCT slices and dentoalveolar variables measured in the upper permanent first premolar region (A), in sagittal view, the reference line (vertical) was positioned in the long axis of the root canal (B) with the result from the axial and sagittal positioning; a coronal view was obtained (C) section for analysis of dentoalveolar variables (D) diagram illustrating the measurements taken (BL, BLC, BT, TL).

After standardization of the CBCT images and tooth positioning, different views were used for analysis. In the axial view, a coronal reference line was positioned in the centre of the crown of the upper first premolar, ensuring it crossed the centre of the root along its long transverse axis (buccal‐palatal) (Figure 1A). In the sagittal view, the mesio‐distal inclination of the first premolar was adjusted so that the coronal reference line passed through the centre of the root along its long axis (Figure 1B). In the coronal view, the upper first premolar was positioned with a line parallel to its long axis, using the buccal cusp tip and the root apex as reference points (Figure 1C).

The measurements of alveolar bone level and buccal thickness were conducted after standardizing the images in the coronal aspect. Full‐screen visualization mode was used for identifying reference points and taking measurements. Magnifying the image, the cementoenamel junction (CEJ) and the buccal alveolar bone crest were identified. The bone level and thickness were measured on the buccal aspect, with the bone level assessed in two distinct ways, depending on the reference used: CEJ (bone level [BL]) or cusp tip (Bone Level to the Cusp Tip [BLC]), as illustrated in Figure 1D. In the same coronal section, the alveolar bone thickness was evaluated at a height of 5 mm from the CEJ in an apical direction. With the coronal image enlarged, a horizontal reference line was positioned over the buccal CEJ. Subsequently, at this level, a new line was drawn to measure the distance between the outer buccal root surface and the outermost portion of the alveolar bone thickness (BT) (Figure 1D).

All measurements of bone level and thickness were performed on both right and left upper first premolars, and are summarized as follows: (Figure 1D)

- *BL*: Distance from the CEJ to the most cervical portion of the alveolar bone crest on the buccal surface of the upper first premolar.
- *BLC*: Distance from the buccal cusp tip of the first premolar to the alveolar bone crest on the buccal surface of the upper first premolar.
- *Alveolar BT*: Distance between the outer surface of the first premolar and the outer surface of the buccal cortical, measured 5 mm from the CEJ.

### 2.3 Dental Measurements—Upper First Premolars

In addition to evaluating alveolar bone level and thickness, measurements were taken to quantify the transverse increment and dental inclination in the region of the first premolars (teeth 14 and 24) before and after maxillary expansion.

**FIGURE 2:**
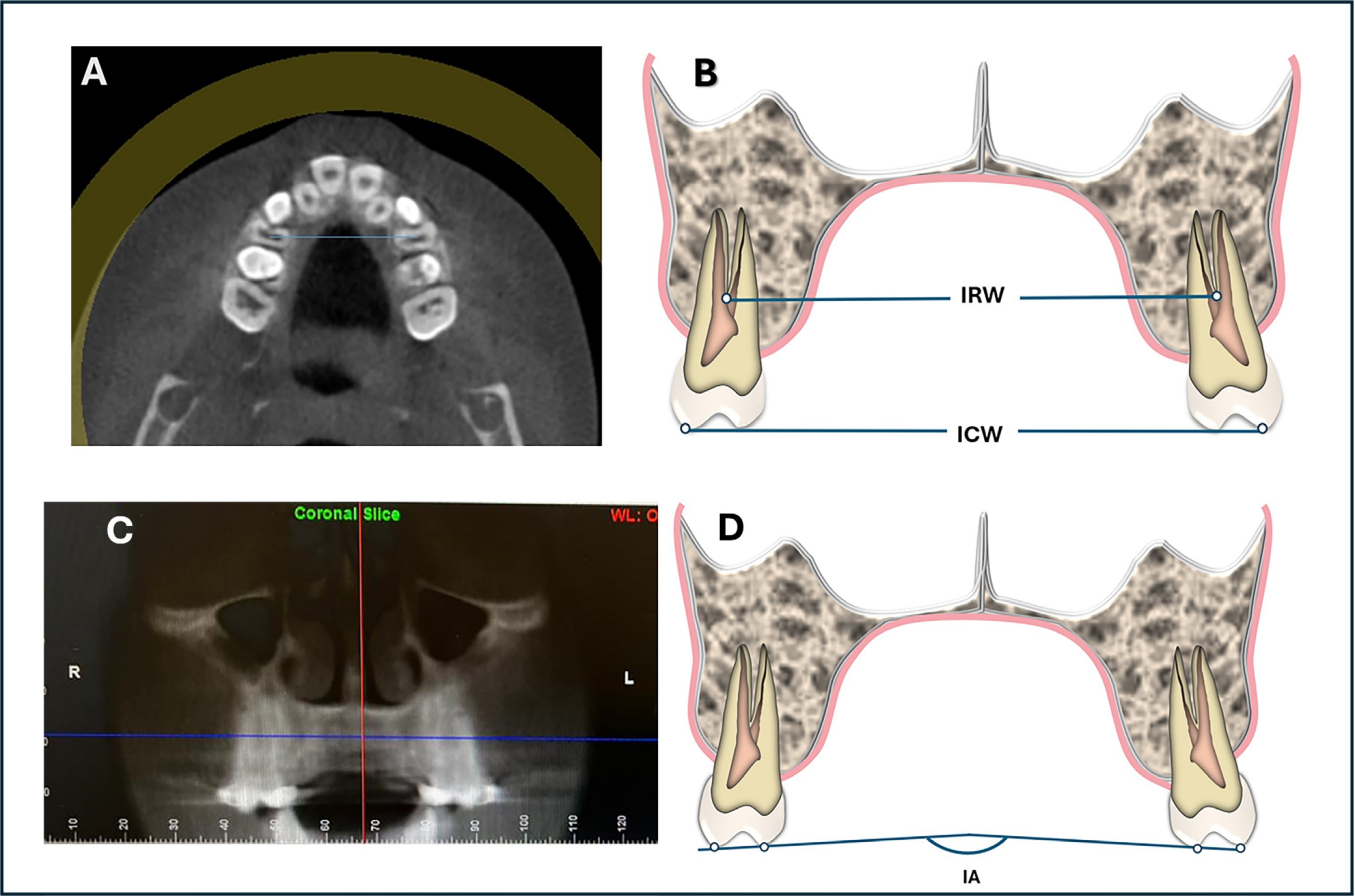
(A) Axial view to determine the points for measuring the distance between the roots of the first premolars. (B) Coronal view in the first premolar region, showing the interradicular distance (IRW) and distance between the cusps (ICW) of the upper first premolars. (C) and (D) image illustrating the angle formed by the inclination of the first premolars (IA), measured after connecting the buccal and palatal cusp tips of the right and left upper first premolars (14 and 24).

Interpremolar distances were evaluated in two vertical levels: at the root and the crown levels. The interpremolars root width (IRW) was measured in the coronal view by determining the following points in the axial view: at the height of the centre of the root canal (for single‐rooted teeth) or the centre of the root furcation (for multi‐rooted teeth), measuring the distance between the right and left sides. The distance between the upper first premolar crowns was measured in the coronal section, in millimetres, between the buccal cusp tips of the upper first premolars (interpremolars cusp tips width—ICW) [18] (Figure 2, A and B).

The Interpremolars Angle (IA) was evaluated in the coronal section, quantifying the dental inclination between the first premolars before and after maxillary expansion. A line was drawn passing through the buccal and palatal cusp tips of the right and left upper first premolars. At the point where the two lines intersected, the angle measurement tool was used (Figure 2, C and D).

For the tooth length (TL) evaluation, the reference was taken from the buccal cusp tip of the crown of the upper first premolars to the root apex (Figure 1D). All measurements were performed blindly by a single researcher (J.P.).

The dental measurements were performed on both right and left upper first premolars and are summarized as follows:

- *TL*: Distance between the tip of the buccal cusp to the apex of the root of the maxillary first premolar (Figure 1D)
- *IRW*: Interpremolars distance measured between the points located at half the height of the root canal (single‐rooted tooth) or in the centre of the root furcation (bi‐rooted teeth) (Figure 2B)
- *ICW*: Distance between the premolars, taking as reference the buccal cusp tip of the upper first premolars (Figure 2B)
- *IA*: Angle formed by the upper first premolars, measured using two lines drawn between the buccal and palatal cusps of the upper first premolars (Figure 2D)

The dental maturation of the upper first premolars (teeth 14 and 24) was assessed according to Nolla's stages using CBCT images obtained in (T1). Evaluations were performed in the axial, sagittal, and coronal views to identify the extent of root formation and apical development (6: Crown completed; 7: One‐third of root completed; 8: Two‐thirds of root completed, open apex; 9: Root almost complete; 10: Apical end of root completed) [19].

### 2.4 Statistical Analysis

Intra‐examiner and inter‐examiner reliability were determined by intraclass correlation coefficients (ICC). Intra‐examiner error (J.P.) was assessed by repeating measurements 7 days apart on 20 randomly selected CBCT images. The inter‐examiner error was calculated after a second examiner (F.A.) carried out measurements on 10 CBCT images, and these were compared to the measurements carried out by the first examiner (J.P.).

Statistical analysis of the data was performed using the SPSS statistical package (version 20.0) and the Microsoft Excel spreadsheet. Categorical variables were described by frequencies and percentages. The normality assumption of quantitative variables was assessed using the Shapiro–Wilk test. Quantitative variables that met the normality assumption were described by mean and standard deviation and compared using the Student *t*‐test for paired samples. Student *t*‐tests for independent samples and analysis of variance (ANOVA) for repeated measures were used for comparison between groups. A significance level of 5% (*p* < 0.05) was considered for the comparisons established. To detect a difference equivalent to one standard deviation in any variable within pre‐ and post‐expansion assessments, assuming a significance level of 5% and a sample size of 20 patients, the study achieved a statistical power of 98.9%. For within‐group comparisons, the estimated statistical power was 80.3%. These power calculations were performed using the online PSS Health tool [20].

## 3 Results

A total of 20 individuals were enrolled in the study, 13 boys and seven girls, with a mean age of 11.7 years (SD = 1.9). The RME group comprised 11 individuals, and the Alt‐RAMEC group comprised nine individuals.

The measurements of all variables were considered reliable. The intraclass and ICC were considered excellent [21], with a mean value of 0.79.

The effect of maxillary expansion on the premolars was evaluated using the Student *t*‐test for paired samples, analysing the variation between the times (T1 and T2) of the measurements. To verify whether the activation protocol made a difference in the upper premolar region, the sample was separated into two groups (RME or Alt‐RAMEC), and the data were analysed separately. The samples from the two groups (RME and Alt‐RAMEC) were similar at T1 when evaluated using the Student's *t*‐test for independent samples. ANOVA for repeated measures was used to compare possible differences in variation between groups. All the results are presented in Table 1.

**TABLE 1:** Effects of maxillary expansion on first premolars. Means and differences for all sample and between groups (RME and Alt‐RAMEC), in T1 and T2.

		T1 mean (SD)	T2 mean (SD)	*p* ^a^ (T1 e T2)	Mean difference (T2−T1) (95% CI)	*p* ^b^ (RME × Alt‐RAMEC)
BL	All Sample	1.63 (0.45)	1.67 (0.63)	0.811	0.04 (−0.29 to 0.37)	
	RME	1.76 (0.49)	1.93 (0.59)	0.453	0.18 (−0.33 to 0.68)	0.351
	Alt‐RAMEC	1.47 (0.38)	1.34 (0.55)	0.572	−0.13 (−0.64 to 0.38)	
BLC	All Sample	8.20 (1.09)	7.75 (1.45)	0.129	−0.45 (−1.05 to 0.14)	
	RME	8.55 (0.97)	8.59 (0.51)	0.873	0.04 (−0.55 to 0.64)	0.051
	Alt‐RAMEC	7.78 (1.13)	6.72 (1.58)	0.061	−1.06 (−2.18 to 0.06)	
BT	All Sample	2.17 (0.63)	1.63 (0.70)	0.012^c^	−0.54 (−0.95 to −0.14)	
	RME	2.36 (0.46)	1.90 (0.54)	0.040^d^	−0.46 (−0.90 to −0.03)	0.670
	Alt‐RAMEC	1.93 (0.75)	1.30 (0.76)	0.124	−0.64 (−1.49 to 0.22)	
TL	All Sample	18.32 (2.45)	18.24 (2.20)	0.872	−0.08 (−1.14 to 0.98)	
	RME	19.10 (2.40)	19.16 (2.17)	0.934	0.06 (−1.29 to 1.39)	
	Alt‐RAMEC	17.35 (2.56)	17.10 (2.93)	0.736	−0.25 (−0.18 to 0.13)	0.739
ICW	All Sample	40.13 (2.67)	45.09 (3.78)	< 0.001^c^	4.97 (3.52 to 6.41)	
	RME	40.15 (1,96)	44.62 (3.00)	0.001^d^	4.46 (2.49 to 6.44)	0.437
	Alt‐RAMEC	40.09 (3.49)	45.67 (4.70)	0.001^d^	5.58 (3.02 to 8.14)	
IRW	All Sample	33.96 (2.50)	38.25 (2.79)	< 0.001^c^	4.29 (3.29–5.29)	
	RME	34.22 (1.89)	38.67 (2.65)	< 0.001^d^	4.45 (2.76 to 6.14)	0.719
	Alt‐RAMEC	33.65 (3.19)	37.74 (3.02)	< 0.001^d^	4.09 (2.78 to 5.40)	
IA	All Sample	161.30 (10.78)	164.95 (6.72)	0.127	3.65 (−1.14 to 8.44)	
	RME	161.83 (11.37)	161.50 (7.11)	0.903	−0.33 (−6.27 to 5.61)	0.051
	Alt‐RAMEC	160.65 (10.66)	169.17 (2.75)	0.036^d^	8.52 (0.73 to 16.30)	

Abbreviation: CI, confidence interval.

^a^ Student *t*‐test for independent samples (*p* < 0.05).

^b^ Repeated measures ANOVA (*p* < 0.05).

^c^ Effect statistically significant for all samples, between T1 and T2.

^d^ Effect statistically significant between groups (RME and Alt‐Ramec) in T1 and T2.

After maxillary expansion, a significant increase in interpremolar distances was observed, both in the IRW and ICW measurements, which increased on average by 4.29 mm and 4.97 mm, respectively. This increased pattern indicated a tilting movement of the premolars, with greater coronal than root distance. Another measurement that showed a statistical difference before and after expansion was alveolar BT. There was an average reduction of 0.54 mm after expansion. The other measurements did not show a statistical difference.

**FIGURE 3:**
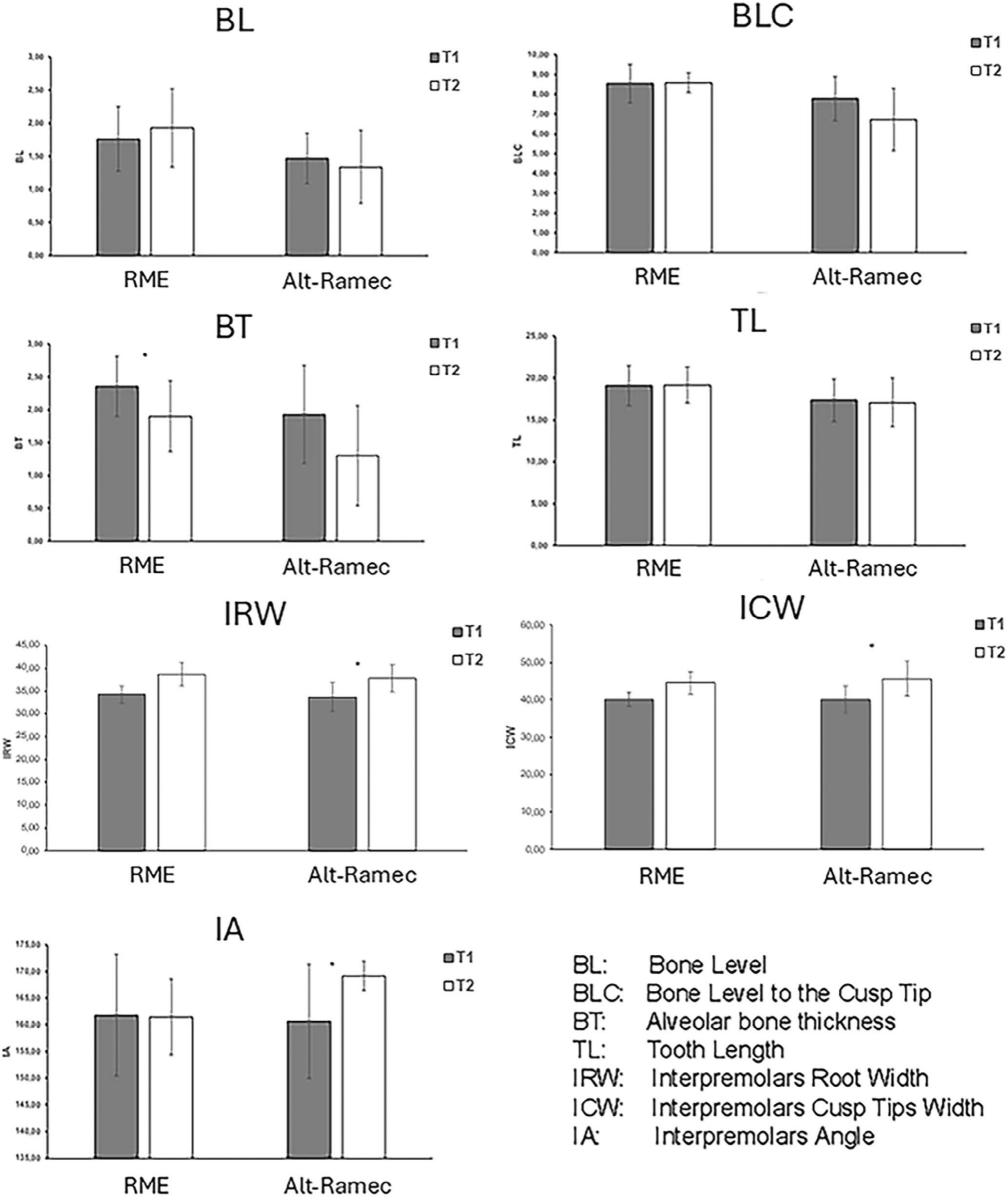
Comparison between groups for each measurement, according to the maxillary expansion protocol (RME or Alt‐RAMEC). The asterisk (*) denotes a statistically significant difference between protocols.

Analysing the RME and Alt‐RAMEC groups individually, it was observed that the BT, ICW and IRW measurements showed a statistical difference in the RME group after T2 (Table 1, Figure 3). Alveolar BT was reduced (0.46 mm), and the premolars were distanced significantly after expansion, with the crown and root moving apart almost the same amount (ICW = 4.45 mm and IRW = 4.46 mm), indicating a more translational movement of the premolars in this group. Regarding the Alt‐RAMEC group, the ICW, IRW and IA measurements showed a significant difference between T1 and T2. There was an increase in interpremolar distances, but with a greater distance from the crowns than the roots (ICW = 5.58 mm and IRW = 4.09 mm), revealing greater tooth inclination. The IA angle corroborates this finding (with an increase of 8.02^o^ in the Alt‐RAMEC group), different from what was observed in the RME group (0.33°).

When comparing the two expansion protocols (RME × Alt‐RAMEC) no statistical differences were observed between them (Table 1).

In addition, the upper first premolar regions were observed and compared (T1 vs. T2) using CBCT, to detect possible anatomic defects in the buccal cortical plates. The findings at T2 were classified accordingly, with or without the involvement of the cortical buccal plates. A total of 40 first premolars were analysed (22 ot the RMEgroup and 18 of the Alt‐Ramec). In the RME group, 17 teeth showed buccal cortical plate involvement, most frequently in the cervical third. In the Alt‐RAMEC group, 13 teeth exhibited involvement, distributed across cervical, middle and apical thirds. In total, 30 teeth showed some degree of buccal cortical plate involvement, while 10 teeth presented no alteration.

The Nolla developmental stages of the upper first premolars (14 and 24) were assessed individually, comprising 18 teeth in the Alt‐RAMEC group and 22 in the RME group. Stages ranged from 7 to 10 in the Alt‐RAMEC group (mean = 8.89; SD = 0.96) and from 9 to 10 in the RME group (mean = 9.50; SD = 0.51).

## 4 Discussion

Several studies have evaluated maxillary expansion in the molar region [9, 15, 16, 22, 23]. However, far fewer investigations have focused on the premolar region [22, 24, 25, 26], particularly using CBCT, despite this area being directly involved in anchorage of the expansion appliances.

In the present study, the dentoalveolar effects of maxillary expansion on the first premolars were evaluated, including interpremolar distance and angulation, tooth length, alveolar bone level and thickness. To our knowledge, this is the first three‐dimensional study evaluating the effects of conventional (RME) and expansion‐constriction (Alt‐RAMEC) maxillary expansion protocols on the premolar region.

The null hypothesis of the present study was that the two expansion protocols (RME and Alt‐RAMEC) would not differ in their effects on dentoalveolar changes in the premolar region. In this study, the mean response to maxillary expansion in the premolar region showed a significant increase of 4.29 mm and 4.97 mm in interpremolar distances, measured at the root and crown heights (IRW and ICW). Thus, a tilting movement of the premolars, with greater coronal than radicular movement, was observed (Table 1). This greater coronal movement was significant in the Alt‐RAMEC group, with a greater distance from the crowns than from the root. Significant crown tipping was not observed in the RME group. It is believed that with the RME protocol, the rapid force application does not allow enough time for tooth movement, so the force is transferred to the sutures, and the sutures open up [27]. On the other hand, in the Alt‐RAMEC group, the one‐week activation‐deactivation protocol may have distributed the forces differently across the teeth and supporting tissues, promoting greater buccal inclination of the premolar crowns. This occurred even though the appliances used in the present study were identical in both groups (Hyrax type appliance), as the double‐hinged screw suggested by Liu and Tsai [6] was not used. The IA angle confirms greater crown inclination in the Alt‐RAMEC group (showing an increase of 8.02° from T1 to T2), in contrast to the RME group, where no significant difference was observed (0.33°). This indicates more translational movement of the premolars in the RME group [22] and greater inclination in the Alt‐RAMEC group.

**FIGURE 4:**
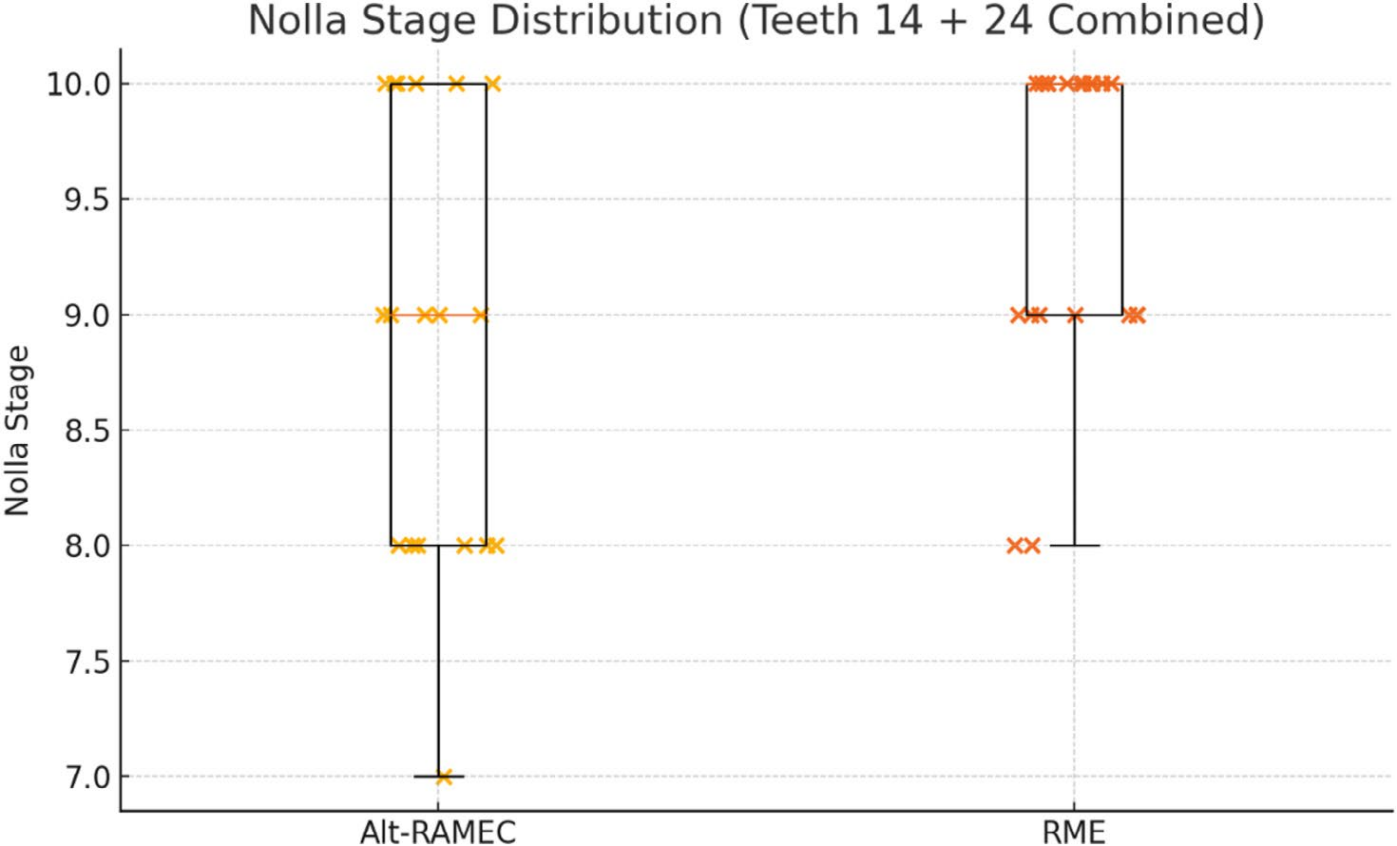
Nolla developmental root stage distribution of the upper first premolars (teeth 14 and 24 combined) for both expansion protocols (Alt‐RAMEC group included 18 premolars, and the RME group included 22 premolars). Each data point represents an individual tooth. Boxplots indicate median, interquartile range and extreme values, while jittered points illustrate the distribution of all teeth within each protocol.

In the present study, the only difference between the evaluated groups was the activation protocol (amount and type of activation), as the patients' ages, Nolla developmental stages and the appliances' design were the same (Hyrax with four bands). The analysis of Nolla's developmental stages indicated that the premolars in both groups were already at an advanced stage of root development at T1, supporting the similarity and comparability of the groups in T1 (Figure 4). Thus, it is believed that the greater dental inclination observed in the Alt‐RAMEC group could be a response of periodontal tissues to the expansion and constriction forces to which they were exposed, resulting in increased dental mobility, which overpowered the anchorage of the appliance. It can be inferred that the unidirectional force of RME promoted expansion with translational movement in the premolar region, while Alt‐RAMEC, due to its intermittent forces, led to greater inclination after expansion rather than translational movement of the tooth. Rinaldi et al. [15] also observed a greater crown inclination, but in the molar region in response to different activation protocols after maxillary expansion.

**FIGURE 5:**
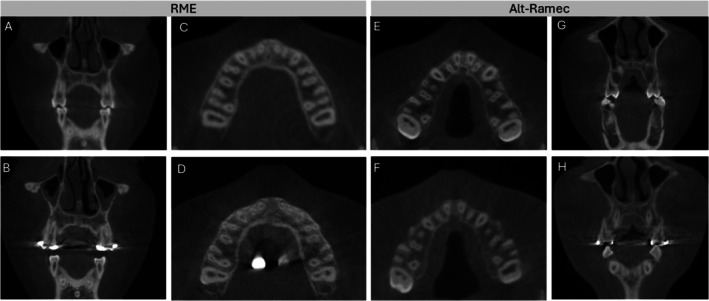
Images illustrating the anatomic evaluation at the first premolars region, axial and coronal views, before (T1) and 6 months after expansion (T2). Observe the transposition of the buccal cortical plate after expansion. RME case (T1: A, C; T2: B, D); Alt‐RAMEC case (T1: E, G; T2: F, H).

A reduction in buccal alveolar BT in the premolar region was observed after the expansion procedure, with an average decrease of 0.54 mm (Table 1). Garib et al. [22] evaluated periodontal changes in CBCT after maxillary expansion using two types of expanders: tooth‐tissue‐borne and tooth‐borne. A reduction in buccal alveolar thickness was observed in the anchorage teeth (premolars and molars), resulting in translational movement of these teeth. The more significant reduction in bone thickness observed in the premolar region could be related to the anatomy, which differs from that of the molar region. The first molar is positioned in an area with a greater bone volume in the apical root region. In contrast, the first premolar is located in an area with less bone volume in the apical region (with a significant narrowing near the root apex). In some patients, the movement induced by the maxillary expansion promoted transposition of the alveolar bone in the first premolar and first molar regions [22]. In the present study, a similar trend was observed using the tooth‐borne appliance and the two different activation protocols, with a decrease in alveolar thickness in the premolar region, and involvement of the alveolar bone in some cases and areas (Figure 5). The reduction in BT measurement was significant for the conventional expansion protocol (RME).

Bone level measurements (BL and BCL) did not show a statistically significant difference between T1 and T2 (Table 1). There was a trend towards a reduction in this measurement in the Alt‐RAMEC group and an increase in the RME group. This distinct response could be associated with the different movement patterns observed in the two groups in the premolar region (more inclination with Alt‐RAMEC and more bodily movement with RME). Nevertheless, the movements promoted by the two maxillary expansion protocols did not cause a significant loss of alveolar bone crest in the premolar region within the 6‐month post‐expansion period.

On the other hand, Rinaldi et al. [15] observed deleterious alveolar effects in the molar region after Alt‐RAMEC. The authors commented that this could be due to greater crown inclination, resulting in bone crest resorption and loss of the periodontal ligament in the anchorage teeth, particularly with the repetitive‐activation protocol, as observed in Alt‐RAMEC, with a rigid appliance like the Hyrax. This group (Alt‐RAMEC) showed greater dehiscence, fenestration and root exposure in the first permanent molars region [15]. The force exerted on the supporting teeth during screw activation induces hyalinization of the periodontal ligament on the pressure side. Initially, necrotic tissue could be favourable, as it could obstruct alveolar bone resorption and consequent tooth movement. If the tooth is unable to move, it transmits the ideal forces for orthopaedic movement. However, the accumulated force is used to promote maxillary rupture at the start of activation. Possibly, orthodontic effects occur subsequently, with the residual force remaining because the orthopaedic effect is less than the total expansion. The negative consequences of periodontal ligament hyalinization can appear, represented by the alveolar bone loss of the supporting teeth and root resorption [28].

The first premolar's tooth length (TL) did not show a statistically significant difference after maxillary expansion procedures. Table 1 showed that there was no loss of root structure in the RME group. The slight increase in this measurement could be attributed to the closure of the root apex, possibly due to the age range of the sample evaluated. In the Alt‐RAMEC group, there was a tendency for a reduction in tooth length (0.25 mm). This reduction could be associated with the type of tooth movement (expansion‐constriction with greater tooth inclination), interfering with the natural process of root apex closure. Evaluating the molar region and comparing different expansion protocols, Rinaldi et al. [15] observed a reduction with significant shortening in the length of the mesiobuccal root of the first molars, except for the Alt‐RAMEC group (with an average reduction of 0.18 ± 0.12 mm). Previous works [25, 29] evaluating root volume, surface changes and root shortening of the first molars and first premolars after RME therapy found a significant loss in their root volume following maxillary expansion.

The upper first premolar regions were observed and compared to detect possible anatomic defects in the buccal cortical plates at T1 and T2. The findings at T2 were classified accordingly, with or without involvement of the cortical buccal plates and the root third involved. The vestibular bone plate was involved in most of the cases (83%). There was greater apical involvement in the RME group, probably due to the type of tooth movement (more translational movement with RME and inclination with Alt‐RAMEC).

A recent retrospective study by Türker et al. [30] compared the effects of RME and Alt‐RAMEC protocols on root development in 40 patients with Class III skeletal malocclusion treated with maxillary expansion followed by Petit‐type facemask therapy. Root lengths of maxillary and mandibular canines, premolars and first molars were assessed using panoramic radiographs before and after treatment. The results showed clinically similar outcomes between the two protocols when age and individual variability were considered [30]. However, the use of two‐dimensional imaging rather than CBCT may limit the precision of root length assessments.

The present study has some potential limitations. The sample was obtained from a well‐defined, methodologically homogeneous cohort derived from controlled clinical trials, thereby helping ensure consistency despite the limited number of cases. The sample demonstrated adequate statistical power to detect significant dentoalveolar changes (98.9% overall; 80.3% within‐group comparisons), supporting the reliability of the observed effects. As a retrospective analysis, the study reflects clinical decision‐making at the time of treatment, which may introduce heterogeneity; however, the outcomes assessed were limited to the expansion phase. Evaluations conducted 6 months post‐expansion may not fully capture the long‐term effects of maxillary expansion, as dental and bone structures can continue to adapt beyond this period. Long‐term evaluations with larger samples are essential to clarify these findings and provide a more comprehensive understanding of the adaptations in tooth inclination and vestibular bone plate thickness following maxillary expansion. Technical limitations of CBCT imaging, such as differences in image acquisition settings [31], resolution and accuracy, could impact the precision of measurements for alveolar bone height and thickness. Although reducing CBCT voxel size could enhance image accuracy for alveolar bone analysis, this approach would increase the patient's radiation dose, which conflicts with the ALARA (as low as reasonably achievable) principles.

The results of the present study corroborate previous findings that tooth‐borne expanders produce some deleterious effects on the anchorage teeth [16, 22, 26, 29], suggesting greater periodontal consequences with RME and dental involvement with Alt‐Ramec at the first premolar region. Although Akyalcin et al. [24] described that alveolar bone changes after RME were reversible in the long term, the loss of tooth volume [22, 24, 26, 29] is not. These findings indicate the importance of performing maxillary expansion as early as possible, during the deciduous and mixed dentition. Using deciduous teeth as anchorage would prevent adverse effects on the permanent teeth, and the periodontal involvement would be overcome with the eruption process of the permanent teeth and alveolar bone formation, reestablishing the area's integrity [22].

Another possible alternative to reduce dental side effects and enhance skeletal outcomes in growing patients is the use of tooth–bone–borne expanders (MARPE). When comparing the dentoskeletal changes produced by a conventional tooth‐borne (Hyrax expander) and a tooth–bone–borne design incorporating four mini‐implants (MARPE), they observed minimal changes in upper first premolar angulation with the tooth‐bone‐borne group. This could be associated with the mini‐implant support that provided a more uniform tension distribution of forces, reducing the dental side effects in this region [32].

It is important to note that the behaviour of each dental group varies and depends on the stage of root formation, tooth volume, region where the anchorage tooth is located (bone quality and quantity), the timing of intervention and force applied (including appliance design, anchorage and activation protocol). A clinical examination considering all these aspects associated with a periodontal risk assessment is recommended. Thus, the findings of the present study highlight the importance of careful protocol selection and anchorage planning to minimize adverse dentoalveolar effects during maxillary expansion.

## 5 Conclusion

– Both RME and Alt‐RAMEC protocols produced significant transverse expansion in the premolar region. The Alt‐RAMEC protocol was associated with greater buccal crown tipping, while the RME resulted in more translational tooth movement.
– A significant reduction in buccal bone alveolar thickness (on average 0.5 mm) was observed in both groups, with no significant changes in alveolar level.
– No significant changes were detected in premolar tooth length. However, a slight reduction was noted after the Alt‐RAMEC protocol, suggesting a potential risk of root resorption in this region when using the alternate expansion and constriction forces with the Hyrax appliance.

## Author Contributions

J.P.: methodology, investigation, writing – review and editing. F.A.: conceptualization, methodology, investigation, writing – review and editing. S.M.D.R: conceptualization, methodology. C.C.A.Q.: writing – original draft preparation, writing – review and editing. T.E.: writing – review and editing. J.M.P.: writing – review and editing. L.M.M.: conceptualization, project administration, funding acquisition, supervision, writing – original draft preparation, writing – review and editing.

## Funding

This study was financed in part by the Coordenação de Aperfeiçoamento de Pessoal de Nivel Superior—Brasil (CAPES)—Finance Code 001.

## Ethics Statement

This study was approved by the Ethics Committee and Institutional Review Board of the Pontifical Catholic University of Rio Grande do Sul (PUCRS) in Brazil (no. 42856915.1.0000.5336). The research was conducted in accordance with the principles of the Declaration of Helsinki. Written informed consent was obtained from the patients and from parents or legal guardians of minors at the time of treatment. The present investigation represents a retrospective analysis of anonymized clinical records.

## Conflicts of Interest

The authors declare no conflicts of interest.

## Data Availability

The data that support the findings of this study are available on request from the corresponding author. The data are not publicly available due to privacy or ethical restrictions.
